# Multiscale perspectives of fire, climate and humans in western North America and the Jemez Mountains, USA

**DOI:** 10.1098/rstb.2015.0168

**Published:** 2016-06-05

**Authors:** Thomas W. Swetnam, Joshua Farella, Christopher I. Roos, Matthew J. Liebmann, Donald A. Falk, Craig D. Allen

**Affiliations:** 1 Laboratory of Tree-Ring Research, University of Arizona, Tucson, AZ 85721, USA; 2 School of Natural Resources and Environment, University of Arizona, Tucson, AZ 85721, USA; 3 Department of Anthropology, Southern Methodist University, Dallas, TX, USA; 4 Department of Anthropology, Harvard University, Cambridge, MA, USA; 5 US Geological Survey, Jemez Mountains Field Station, Los Alamos, NM, USA

**Keywords:** fire history, dendrochronology, archaeology, land uses, Pueblo people, ponderosa pine forest

## Abstract

Interannual climate variations have been important drivers of wildfire occurrence in ponderosa pine forests across western North America for at least 400 years, but at finer scales of mountain ranges and landscapes human land uses sometimes over-rode climate influences. We reconstruct and analyse effects of high human population densities in forests of the Jemez Mountains, New Mexico from *ca* 1300 CE to Present. Prior to the 1680 Pueblo Revolt, human land uses reduced the occurrence of widespread fires while simultaneously adding more ignitions resulting in many small-extent fires. During the 18th and 19th centuries, wet/dry oscillations and their effects on fuels dynamics controlled widespread fire occurrence. In the late 19th century, intensive livestock grazing disrupted fuels continuity and fire spread and then active fire suppression maintained the absence of widespread surface fires during most of the 20th century. The abundance and continuity of fuels is the most important controlling variable in fire regimes of these semi-arid forests. Reduction of widespread fires owing to reduction of fuel continuity emerges as a hallmark of extensive human impacts on past forests and fire regimes.

This article is part of the themed issue ‘The interaction of fire and mankind’.

## 1. Introduction

People living within fire-prone forested environments over long periods of time have profound impacts on forest structures and fire regimes, and vice versa. Prior to the Industrial Age, long-term fuelwood and timber harvesting, agriculture, livestock grazing and the use of trails and roads tended to reduce fuels in woodland and forest landscapes [1,2]. These land uses were especially effective in reducing fire spread in semi-arid landscapes of the south-western USA, where vegetation productivity was relatively low [3–5]. At the same time that fuel connectivity and widespread fires were reduced by human land uses, purposeful and accidental ignitions were added to those occurring from lightning strikes [6]. Hence, there were counteracting effects of human actions on fire frequency versus extent. Myriad human land uses and their effects were superimposed upon broad-scale climate patterns controlling lightning ignition rates, fuel productivity, continuity and moisture content. Climate variability also affected human populations through agricultural productivity and in other ways [7].

Given these complex interactions and their temporal and spatial variability, it is not surprising that it has proven difficult to disentangle the relative roles of human and non-human factors in controlling fire regimes [8]. Although there are extensive historical narratives of human interactions with fire worldwide [9,10], progress has been slow in developing general ecological theory or dynamical models of climate–fire–human interactions (but see recent attempts to derive various foundational concepts: [11–17]).

One of the difficulties in developing and testing general theory is a lack of sufficiently detailed and long-term chronologies of the key variables from the same landscapes. In particular, assessments of patterns and associations in forest ecosystems require multi-century, high-resolution chronologies of precipitation, temperature, numbers of fires, area burned, human population sizes and land uses. There are many examples in the palaeo-ecological literature where these interactions have been interpreted, but there are relatively few cases where long-term, seasonal to annual resolution records of most or all key variables were simultaneously compared [18–23].

The importance of improved understanding of these dynamics has increased as very large, high-severity wildfires (more than 200 000 ha in some cases) are increasing in some parts of western North America and elsewhere, with substantial and growing impacts on forest ecosystems and people living within them [24–26]. Scientists, fire managers and policy-makers in the USA have recognized the role of human-caused forest structure (fuels) changes in many western forests, as well as the role of warming temperatures and drought [27–30]. Although there is a general consensus among forest and fire ecologists that wildfire trends are related to increases in both fuels and drought magnitude, there is some debate about the relative roles of past fire suppression leading to fuels increases, or changes in fire-fighting tactics versus climatic variability. Some have argued, for example, that recent large, high-severity wildfires in semi-arid ponderosa pine forests of the western USA are not driven by past fire exclusion and fuels changes. Further, it is claimed that these events are within the ‘historical range of variability’ in this type, and that there is no ecological basis for forest restoration aimed at reducing fire severity [31,32]. This remains a minority viewpoint, however, with a large body of ecological literature extending back to the 1950s demonstrating human-induced forest and related fire regime changes in semi-arid forests of western North America [33–40]. There are also multiple rebuttals to the recent interpretations that modern changes in fire regimes are unrelated to human-induced forest changes [39,41–47].

Although the general understanding is robust that both human-induced changes in fuels and warming climate are key drivers of recent wildfire trends in some regions and forest types, it is notable that some Native American communities persisted for centuries within fire-prone forests during major droughts. In contrast, modern communities in many of these same landscapes are now experiencing catastrophic events, destroying hundreds of homes, and in some cases resulting in major type changes in vegetation, extreme post-fire geomorphic responses and other unsustainable changes [25,30,48,49].

Here we evaluate climate and human controls over past fire regimes using recently compiled networks of fire scar-based chronologies from three spatial scales: (i) a subcontinental region of western North America, (ii) landscapes within the Jemez Mountains, a large mountain range in north central New Mexico, USA, and (iii) forest stands within areas of different human land-use intensities and timing within the Jemez Mountains. The Jemez Mountains is especially useful as a landscape scale exemplar of human–fire–climate interactions. Here, human populations—specifically the Jemez, a Towa-speaking Pueblo people—have lived within upland forests and woodlands in relatively high densities. We estimate that 5000–8000 people lived within this area from *ca* 1300 to 1640 CE [50–52]. There were at least 10 large villages of more than 500 rooms each and at least 2700 small, 1–3 room houses (in ruins now) distributed over this area (about 500 km^2^) at *ca* 1600 CE. This density of human occupation would easily qualify as a so-called wildland urban interface under modern definitions in the USA (i.e. 6.17 housing units km^−2^, [53]). The spatial and temporal variability of high, medium and low human population densities and land uses in the Jemez Mountains provides a unique opportunity to evaluate fire regime responses with an especially well developed set of climatic, human and fire chronologies spanning the past 700 years.

## 2. Study area/methods

**Figure 1.**
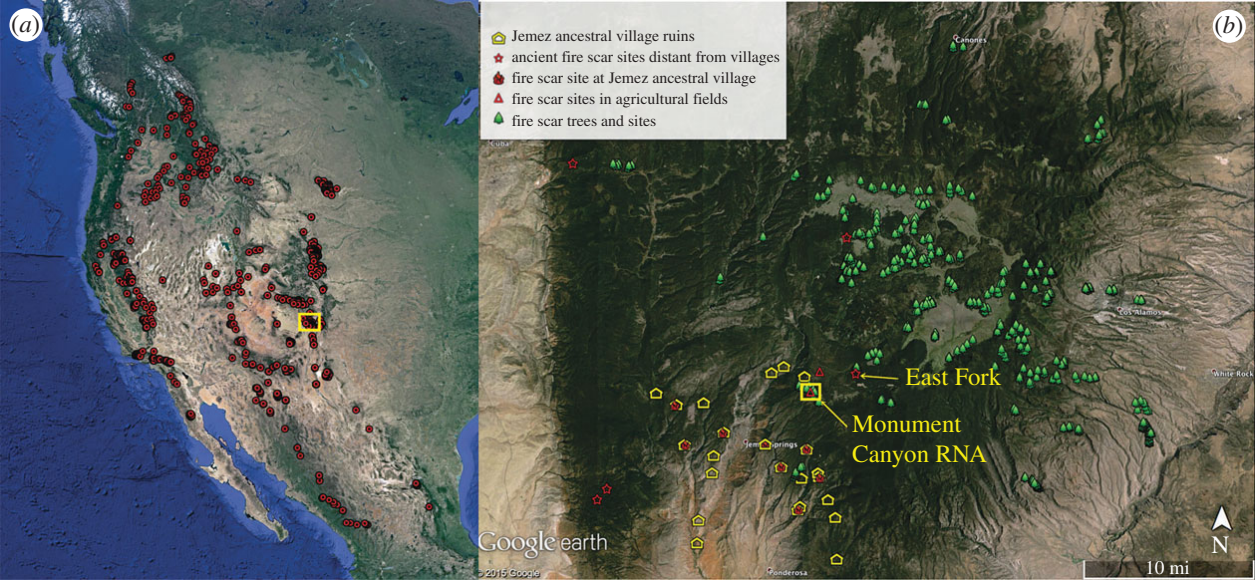
Map of western North America (*a*) shows locations of tree-ring-based fire chronologies (red dots). Sites typically range from 10 to 100 ha in size (forest stands) with 10–30 fire-scarred trees sampled (see electronic supplementary material, figure S1), but some sites are larger (100–1000 ha) with more than 100 trees sampled. The yellow box on the western North America map is the Jemez Mountains of north central New Mexico, shown in the map (*b*). Sampled fire-scarred trees and sites are indicated by symbols and are labelled in the legend. The example sites from a seasonally used agricultural site (Monument Canyon Research Natural Area) and a site distant from villages or agricultural areas (East Fork) are labelled.

For broad-scale context, we use the largest network of tree-ring-based fire scar chronologies in the world to assess interannual fire–climate relations at the subcontinental scale. This data network has recently been compiled from western North America and it provides dates and estimates of the relative extent of fires within forests and woodlands (figure 1 and electronic supplementary material, figure S1 [54]). These data are from more than 800 forest stands and landscapes (during the period of our analyses, 1500–2000 CE), spanning approximately 4.1 million km^2^. Fire-scarred trees have been widely used in North and South America, Scandinavia, and parts of Eurasia to reconstruct multiscale histories of surface, mixed-severity and crown fire regimes (see Falk *et al*. [55] for a review of applications and examples, and see other papers [56–58] for detailed testing of the accuracy and resolution of surface fire history methods as applied in this paper).

We also use one of the largest mountain range-scale networks of fire scar chronologies in existence from the Jemez Mountains of New Mexico. This network comprises 1377 trees collected over an area of about 180 000 ha. Most of these collections are from ponderosa pine-dominated forests (*Pinus ponderosa*), but other forest types and woodlands are also included (e.g. mixed-conifer forests, containing *Pinus*, *Abies* and *Pseudotsuga* species, and spruce-fir forests of *Picea* and *Abies* species). Although these chronologies primarily reflect frequent, low-severity surface fire regimes, they also include so-called mixed-severity and high-severity fire regime types. Comparative studies of high-severity, stand-replacing fire event chronologies at landscape and regional scales with low severity, surface fire event chronologies show that, in general, the large fire years tend to correspond between these types. That is, regionally synchronous high-severity fire years are a subset of the regionally synchronous low-severity fire years [5,39,59,60].

The Jemez Mountains fire scar network includes fire scar collections from three types of sites where human occupation (population) density was relatively high, medium and low, respectively, during the pre-1680 Pueblo Revolt period (back to about 1300 CE in the earliest fire scar records). The high-density sites were large village areas (villages with more than 500 rooms, 3–4 story room blocks and now in ruins) which were generally occupied until the early- or mid-1600s. The medium density sites were agricultural areas with only seasonal use and small houses (1–3 rooms, known as ‘fieldhouses’), and the low-density sites were forest stands relatively distant from both large villages and seasonally used agricultural areas. We used *terminus ante quem* methods to establish or refine human occupation chronologies by crossdating innermost rings of living and dead trees to determine the earliest establishment dates of trees on and near village footprints and agricultural areas following human de-population (see electronic supplementary material, figure S2 and S3 and [61]). The occupation histories of village sites were also determined from ceramics-based chronologies, tree-ring dating of surviving roof timbers and documentary evidence [50–52,61].

Further description and analyses of the large western North American fire chronology network is underway (see [54] for a preliminary description), and most of these chronologies are deposited in the International Multiproxy Paleofire Database. For the purposes of this study, we focus on only the broadest, common patterns across the entire network, and relationships with independently developed drought indices. The reconstructed drought time series are from the North American Drought Atlas project [62]. These records are tree-ring width-based reconstructions of the summer (June, July, August) Palmer drought severity index (PDSI). The Atlas is composed of a set of evenly spaced grid points (separated by 2.5 degrees latitude and longitude) spanning the continent, with a PDSI time series available from each grid point covering periods from *ca* 1 to 2004 CE (the grid point time series vary in length within this period).

We used a drought area index (DAI) compilation of the PDSI data for our assessment of climate–fire relations at the western North American scale in a superposed epoch analysis (SEA). The DAI time series [62] is the percentage of PDSI grid points with annual values less than −1.00 (i.e. all droughts of moderate to severe magnitude) over the western states for each year of the record. Hence, this is an area-weighted estimate of drought magnitude over this broad region of western North America. A declining number of sites have old enough trees to represent comprehensive spatial patterns in earlier times, so we extended our analyses only back to 1600 CE at this scale. For climate–fire analysis of the Jemez Mountains fire scar chronology network, we use a recent tree-ring width-based October–June (i.e. the cool season, plus spring) precipitation reconstruction developed specifically from trees growing within the Jemez Mountains [63]. Using SEAs, we tested the relations between the largest and smallest fire years at the scales of the entire western North American fire chronology network, the entire Jemez Mountains network, and at finer spatial scales in two example forest stands from the Jemez Mountains. The SEA that we employed computed the average climate (DAI and October–June precipitation) conditions (and departures from averages) during the largest and smallest fire years during the entire period or subperiods of time, as well as lagged years prior to and following these years. The SEA uses a bootstrap method to estimate significance of the wet/dry patterns in each of the lagged years and the fire event year [64,65]. We used the 30 largest and smallest fire years in the fire scar network, as estimated by percentages of sites or trees recording fires each year during the period 1500–1860 CE in the Jemez Mountains, and during subperiods before and after the Pueblo Revolt of 1680 CE. The pre- versus post-1680 periods in the Jemez Mountains represent a major change in human population density in the upland areas of this mountain range [52].

Last, we compared fire frequency variations between different historical time periods at the different spatial scales of analyses. To account for changes in sample sizes between periods and potential effects on fire frequency estimates, we used a bootstrap resampling of the master fire chronologies, assessing fire frequency variations as a function of sample size. A more detailed explanation of this method and results of these assessments are in electronic supplementary material, figure S6. We used the software program called FHAES to carry out the SEA, the sample size/fire frequency assessment, and to produce initial versions of the fire scar chronology graphics [66,67].

## 3. Results

### (a) Western North America: broad-scale patterns

**Figure 2.**
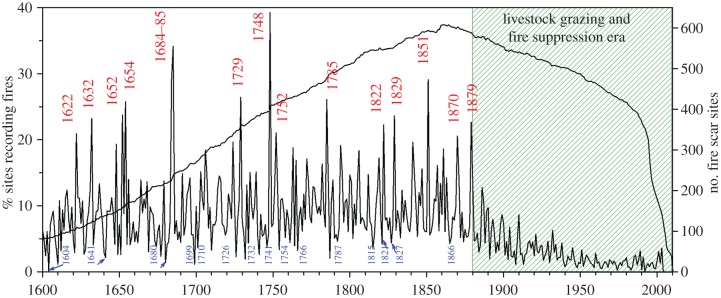
The combined record of fire occurrence from more than 800 sites in western North America shows relatively high fire frequency prior to *ca* 1900, and a high degree of synchrony in both large and small fire years. The 15 largest and smallest fire years are labelled. A pronounced decrease in fire frequency occurred at the time of Euro-American settlement, coinciding approximately with the arrival of railroads, intensive livestock grazing, removal of many Native American populations, and subsequently organized and mechanized fire fighting by government agencies.

The western North American fire chronology network shows a strong pattern of synchronous, large and small fire years extending back to at least 1600 CE (figure 2). Overall, the synchrony of large and small fire events is quite remarkable; the most common fire dates were recorded in more than 25% of the 800 sites, and the largest fire year, i.e. 1748, was recorded in nearly 40% of all sites. The chances of obtaining this degree of synchrony among this number of random time series of events and frequencies are exceedingly small. The strong coherence of the fire occurrence signal across this large portion of western North America demonstrates the high degree to which interannual climate patterns are controlling fire activity. There are no other known environmental variables operating at this spatial extent and long time period that could drive such a high degree of fire occurrence synchrony across forests occurring in separate mountain ranges.

**Figure 3.**
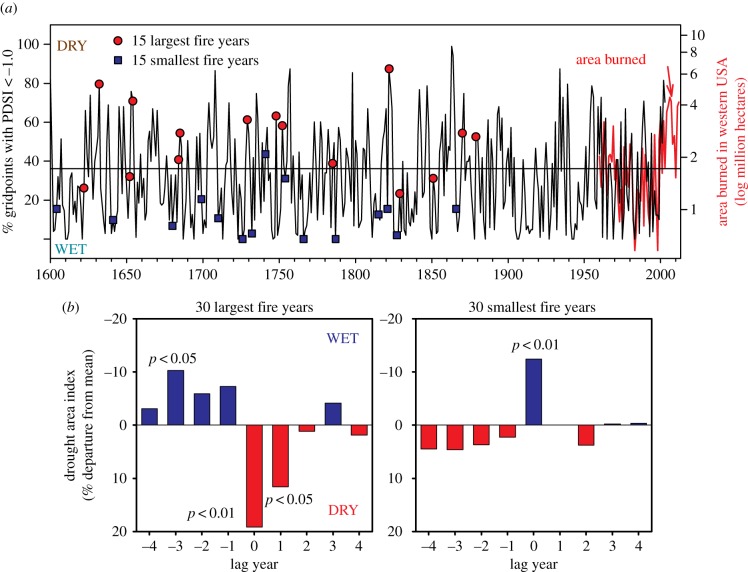
(*a*) Drought area index over the western USA is significantly correlated (*r* = 0.51, *p* < 0.01) with percentage of sites scarred per year over the region and modern area burned in the USA since 1960 (red line below, *r* = 0.37, *p* < 0.01). The 15 largest and smallest fire years are superimposed on the PDSI time series. (*b*) The SEA shows a strong wet/dry pattern associated with large fire years (left), with only wet years being associated with small fire years (right).

As demonstrated in similar studies at finer spatial scales [23,68–70], the highly synchronous high/low fire years in the western North American fire chronology network are well correlated with independently derived interannual climate time series from the same region (figure 3*a*). Moreover, there is also a weak but significant correlation (Spearman rank *R* = 0.37, *p* < 0.01, as also shown in figure 3*a*) of the fire scar-based chronology with modern area burned time series from the western USA, as recorded by government agencies in the period of overlap (1960–2003, figure 3*a* and see [71] for a similar comparison for subregions of the western USA).

The SEA shows a strong wet/dry pattern in the compilation of largest fire years spanning this large region of western North America (figure 3*b*). The pattern of several years of wetter than average conditions preceding a dry year in which widespread fire events occurred has been observed in many ponderosa pine-dominant forests in the western USA ([65] and see examples cited in the previous paragraph). Conversely, smallest fire occurrence years are typically wet, and usually showing no association with conditions during prior years, as evident in the overall western North American network (figure 3*b*). The wet/dry switching of climate conditions in association with the largest fire years is interpreted to be a reflection of the importance of fine fuel production (i.e. tree needles, leaves and grasses) in these frequent surface fire regime forests. This pattern is probably related in part to switching between the wet/dry conditions of western North America owing to La Niña/El Niño states of the Pacific Ocean [72]. This pattern was robustly demonstrated in multi-century-length tree-ring studies [73,74], and is also evident in shorter-term, modern analyses of area burned records in the western USA [75,76]. The wet/dry pattern in association with extensive fires appears to be strongest in lower elevation ponderosa pine forests and in semi-arid grasslands of the Great Basin and south-western regions, where grasses are a critical component of fire ignition and spread dynamics [75,77,78]. The wet/dry pattern tends to be weak or non-existent in higher-elevation, more mesic forests, e.g. mixed-conifer and spruce-fir forest types, where only a strong dry, current year (fire year) signal is typically observed [39,65,79].

Overall, the wet/dry switching pattern as expressed over multiple centuries and large spatial extents is an important clue and indicator of the sensitivity of these systems to fuel continuity/connectivity. Wet prior years leading to increased fine fuel production and high spatial connectivity is critical for widespread fire occurrence during drier than average years. Likewise, the rapid elimination of widespread fires in pine-dominant forests in many locations of the western USA at the time of introduction of intensive livestock grazing during the late 19th century also points to the importance of fine-fuel connectivity. Very large herds of sheep and cattle were introduced in many lowland and upland areas after the railroads were built [80], with a close timing in cessation of widespread fires in many different examples in south-western mountain ranges [3,4,81], including within the Jemez Mountains [82–85]. As such, changes in frequency of widespread fires in ponderosa pine forests emerges as a useful indicator of intensive human land-use changes affecting fuels connectivity, as will also become apparent in analyses of fire extent patterns in the Jemez Mountains long before the introduction of intensive livestock grazing by Europeans colonists.

### (b) Jemez Mountains: medium and fine scales

**Figure 4.**
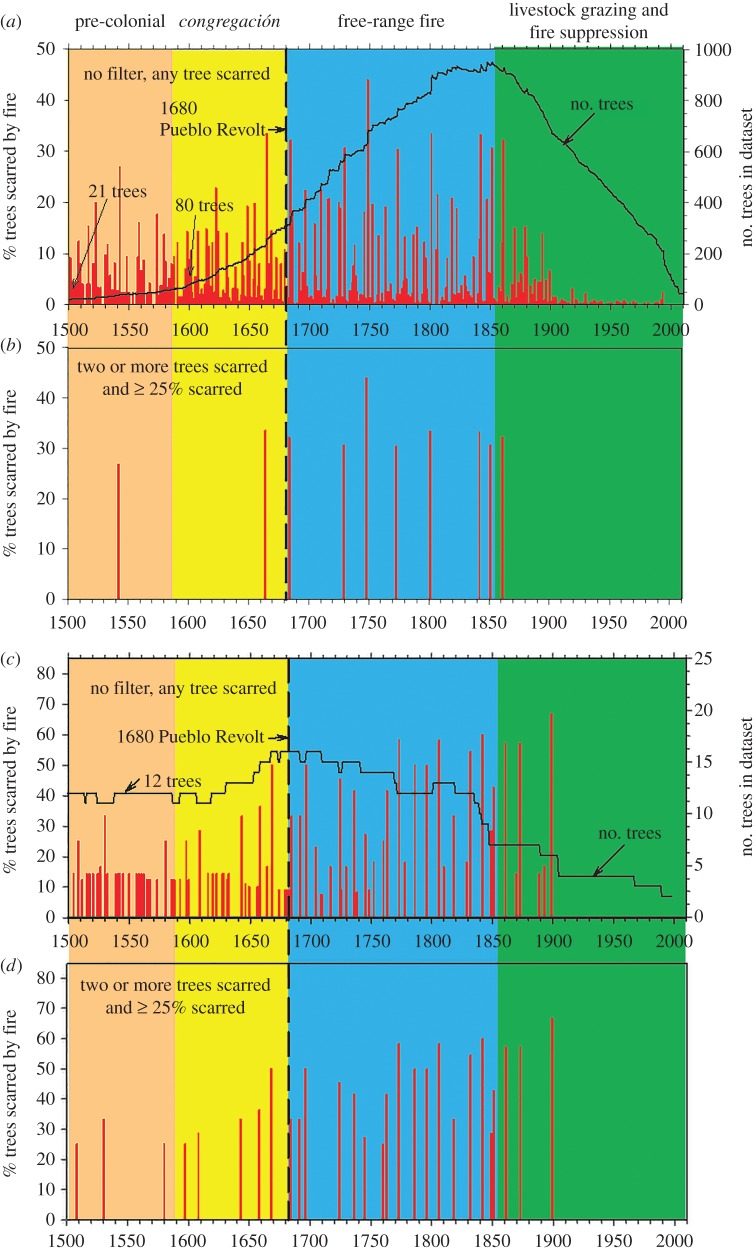
The combined fire scar record from the entire Jemez Mountains (180 000 ha) shows both widespread fires and small fires, as inferred from high/low percentages of trees scarred each year, respectively (*a*). Relatively few widespread fire events occurred prior to 1680 and after 1860 CE, as emphasized in the filtered time series (*b*) that includes only fires recorded by at least two trees and 25% of recording trees. A similar pattern is evident at the fine spatial scale of the East Fork site (5 ha), where sample sizes remained relatively constant from 1500 to 1800 CE (*c,d*).

The Jemez Mountains collection of fire-scarred trees shows an even greater degree of synchrony of large and small fire event years than in the western North American network (in which the Jemez Mountains data are included). The largest fire years in the Jemez, for example, were recorded by 30% or more of the sampled trees, and as high as 44% for the largest fire year in 1748 (figure 4*a*). This is not surprising given there is greater coherency of interannual climate patterns over the relatively smaller regional scale of the Jemez Mountains versus the subcontinental scale of western North America. High synchrony is also owing to the scale and connectivity of the Jemez Mountains, where fires could spread readily between trees and stands [82–85]. In contrast, many of the western North American network sites were separated by deserts, canyons, rivers and great distances, which inhibited fire spread between sites.

A clear pattern of reduced widespread fires was evident during two time periods in the Jemez Mountains network: prior to *ca* 1680 and after *ca* 1860 CE (table 1 and figure 4*a,b*). The post-1860 decline in widespread fires was probably caused by the rise of intensive sheep grazing. Land-use histories of the Jemez region discuss the fact that grazing of upland areas was limited prior to the military subjugation of the Navajo people by the US Army in the mid and late 1860s [80]. Prior to that time, raiding and the theft of livestock and killing of herdsmen were common, resulting in low or no livestock grazing in the interior portions of the Jemez until sometime after the late 1860s. Also, as noted earlier, the arrival of railroads for shipping livestock (after the late 1870s) resulted in great expansion of sheep herds, especially in the great *valle* grasslands of the Jemez Mountains. After World War I, the sheep markets declined, but government fire fighting became more active and effective. Grass fuels were mostly removed by intensive livestock grazing, and landscape-scale discontinuity of fuels was created by the proliferation of ‘driveways’ used for seasonal movement of thousands of sheep up and down the mountains (transhumance), and the daily back and forth herding to water sources [6,81,83].

**Table 1.** Number of fires during periods before and after the 1680 Pueblo Revolt (*n* = 181 years for 1500–1680, *n* = 180 years for 1681–1860) for three spatial scales in the Jemez Mountains. Fire dates recorded by any tree in the composite chronology (all fires), fires recorded by only a single tree (one tree) and fires recorded by 25% or more of all trees are listed for each spatial scale. The statistical significance of a test of differences between the mean fire intervals in the two periods (using the non-parametric Mann–Whitney rank-sum test) is shown in the column on the far right (prob.).

		intervals between fires (years)						
	area (ha)	1500–1680			1681–1860			
	trees sampled	*n*	mean	median	*n*	mean	median	prob.
Jemez Mountains	180 000 1377							
all fires		147	1.25	1	173	1.05	1	0.001
1 tree fires		44	4.10	3	22	8.95	5	0.019
>1 tree fires		105	1.76	1	153	1.19	1	0.001
≥25% fires		2	122.00	122	9	27.83	27	0.001
Monument Canyon	260 198							
all fires		50	3.57	3	70	2.57	2	0.152
1 tree fires		26	6.28	5	15	11.14	4	0.825
>1 tree fires		24	6.48	3	54	3.26	3	0.343
≥25% fires		9	19.13	18	26	13.20	10	0.199
East Fork	5 26							
all fires		46	3.98	4	29	6.07	7	0.002
1 tree fires		37	4.86	4	5	16.75	15	0.003
>1 tree fires		9	21.00	16	24	7.26	8	0.001
≥25% fires		8	21.00	16	24	7.26	8	0.001

The other period showing reduced widespread fires was during the pre-1680 period (table 1 and figure 4). In August of 1680, the Puebloan people of the south-west rose up in a coordinated revolt against the Spanish colonists, who had held the region under an authoritarian regime, subjugating and taxing the native populations since the late 1590s [51,80]. A severe and sustained drought occurred in the 1580s, and then again in the 1660s–1670s [28,62,63], which also undoubtedly placed additional stress on indigenous populations. Introduced diseases and conflicts with the Spanish colonists and with various other groups (and especially with Navajo, Apache and Utes) also drastically reduced Puebloan populations in the south-west. By the late 1600s, populations were reduced by more than 80% [52]. From the Pueblo Revolt of 1680 until the early 1690s, Spanish colonists were absent from northern New Mexico, and after the *Reconquista* of the 1690s they returned. By about 1700 CE, no permanent settlements of Jemez people remained in the uplands of the Jemez Mountains [50–52].

We now have relatively detailed, site-specific chronologies of human presence and land uses in the Jemez Mountains, because recent studies have compiled archaeological and documentary sources in considerable detail [50–52,61,86]. We employed extensive surface ceramics analysis and *terminus ante quem* tree-ring studies to determine human occupation chronology on the same sites where we conducted tree-ring-based fire history analyses (electronic supplementary material, figure S3; [61]). Until the 1590s–1620s CE, when the first Catholic mission churches were built in the Jemez Mountains, the Jemez people occupied at least half a dozen large villages (from 500 to 1500+ rooms each) and many smaller villages within forests and woodlands on *mesa* tops of the southern Jemez Plateau. They had lived within some of these villages and forested landscapes for 200–300 years or longer. The upland village sites were depopulated by the early to mid-1600s owing to a combination of declining populations caused by disease and conflict, and to the forced or unforced congregation (‘*congregación*’ in Spanish) of Jemez people at Catholic missions in lower elevations [50–52,86], and see electronic supplementary material, figure S3).

Tree age structure and fire scar chronologies developed from specimens taken directly on and near a set of large village ruin sites in the Jemez Mountains generally show a lack of spreading fires within or immediately adjacent to the villages during the occupation periods (i.e. pre-1650s, see electronic supplementary material, figure S3; [52,61]). This stands to reason, because the occupation of the village areas by hundreds of people for multiple centuries (in some cases) would have left little or no continuous fuels available in the vicinity for fire ignitions and spread. The largest village sites in the uplands are all above 2100 m in elevation with relatively cold winters, requiring substantial amounts of fuelwood for heating and cooking. Moreover, many thousands of timbers were used in construction of the large, multi-story room blocks, as well as for other purposes. As a consequence, the village footprints were probably essentially treeless during occupation. We generally observed trees recruiting on the village sites one to several decades after the likely departure of most or all people from the site, as corroborated by independent documentary evidence of depopulation of one of the villages at the time of the Reconquest in the early 1690s [52,61]. Fire scars also begin to be recorded on the post-occupation trees after one to several decades, indicating that widespread fires became possible on the village sites once people had left and continuous fuel layers established (electronic supplementary material, figure S3; [52,61]).

At sites relatively distant from villages, where seasonal agriculture was practiced and small house sites (‘fieldhouses’) are commonly present, we detect a different pattern. Many small fire events (i.e. non-synchronous fire scar dates among sampled trees) and relatively few widespread fires occurred prior to 1680 (e.g. at Monument Canyon Research Natural Area, table 1, electronic supplementary material, figure S4 [85]). Many more widespread fires (i.e. synchronous fire events, as estimated by greater than or equal to 25% of trees scarred per event within a sampled site) occurred after *ca* 1680 CE than before. In the Jemez-wide fire scar chronology compilation, only two widespread fires were recorded from 1500 to 1680 CE (181 years), whereas nine widespread fires were recorded from 1681 to 1860 CE (180 years, table 1 and figure 4*a,b*).

A similar pattern is evident in sites that were relatively distant from both villages and seasonal agriculture and fieldhouses, and where we have been able to find ancient fire-scarred tree specimens (table 1 and figure 4*c,d*). Perhaps in these marginal sites, which tend to be rocky and steep, more trees survived fuelwood gathering and timber harvesting during the major occupation period by Jemez people (i.e. *ca* 1300–1680 CE) than in places closer to the villages and agricultural sites. In these locations the resinous, basal portions of long-dead fire-scarred trees persisted on the ground as logs because of rocky and dry conditions, slowing decay processes and protecting the logs somewhat from surface fires. Hence, we were able to find, sample and reconstruct unusually long fire scar chronologies from these old specimens. Figure 4*c,d* shows the 1500–2010 CE portion of one of these sites named ‘East Fork’ (see map in figure 1), and a more detailed presentation of the individual tree records extending back into the 1300s CE from this site is in the electronic supplementary material, figure S5.

Although the Jemez-wide fire chronology (figure 4*a,b*) and the Monument Canyon site chronology (electronic supplementary material, figure S4) show a general decline in sample sizes prior to 1700 CE, the East Fork site sample size decreases less abruptly before this time, with at least 11 trees in the data set back to about 1500 CE (figure 4*c,d* and electronic supplementary material, figure S5). In addition, fire scars are recorded on four or more trees at the East Fork site back into the 1300s CE. This chronology shows the same general pattern as the Jemez-wide and Monument Canyon chronologies with many small fires recorded during the pre-1680 period, but relatively few widespread fires during this period compared with after 1680 CE (table 1). Further, we assessed fire frequency changes over the entire Jemez Mountains, and separately within the Monument Canyon and East Fork sites, while also accounting for sample size effects through time (see supplementary material, figure S6). We found that, in general, the patterns identified in these areas (i.e. few widespread fires prior to 1680 and more small fires prior to 1680 than later) are consistent, even when accounting for sample size changes between periods (electronic supplementary material, figure S6).

**Figure 5.**
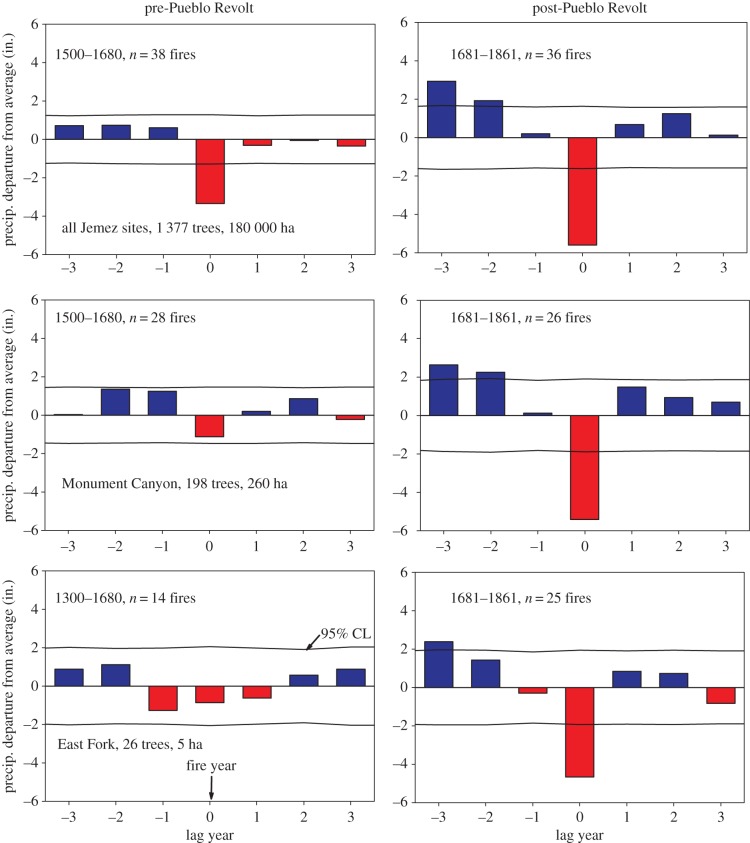
SEA using a tree-ring width-based precipitation reconstruction from the Jemez Mountains [63] and largest and smallest fire event years at three spatial scales in the Jemez Mountains. There is a weak or non-existent relationship between largest fire years' and current or lagging years' moisture prior to 1680, but a strong wet/dry pattern afterwards. Human influences on fire timing probably overrode climate influences prior to 1680.

One additional analysis illustrates important differences between the pre- and post-1680 periods: an SEA of climate–fire associations (figure 5). The results for the Jemez Mountains-wide chronology show that prior to 1680 there is only a weak association between fires and interannual climate (cool season precipitation, i.e. prior October to current June rainfall) variations. Dry conditions were typical during fire years (as recorded by two or more trees), and there was the typical and strong wet/dry pattern of association with fire occurrence after 1680. The seasonal agricultural/fieldhouse site at Monument Canyon site, and the distant, ancient fire scar site at East Fork, show no association with fire events (recorded by two or more trees) prior to 1680, but a significant (*p* < 0.05) wet/dry association with fire after 1680 is evident (figure 5). These findings point to the likely overriding role of human-set fires and disruption of fuels continuity in the pre-1680 period relative to the post-1680 period, when, in the absence of extensive human land uses, interannual climate variations were more dominant.

## 4. Discussion and conclusions

Both the broad-scale, Jemez-wide fire occurrence record and the finer-scale example from Monument Canyon Research Natural Area show general patterns of reduced frequency of widespread fires prior to the 1680 Pueblo Revolt with many small (low-synchrony or single-tree) fire events occurring in this earlier period. It is notable that the relatively low number of widespread fires pre-1680 and high number of low-synchrony fire events (small fires) during that period coincides with decreased numbers of samples in the Jemez-wide and Monument Canyon datasets. This so-called fading-record problem [87] potentially biases the observed fire occurrence patterns in the pre-1680 period. However, the expected effect of sample sizes in composite fire scar chronologies is that fewer fire dates will be recorded overall with smaller sample sizes, and fewer small fires will be detected in particular. That is, as sample sizes (and amount of area sampled) increase, the odds of detecting more fires in the sampled fire-scarred trees increases [58,85]. The pattern observed in the Jemez, however, was the opposite of this expectation. That is, there were actually more small-fire events (single-tree fire events) recorded in the pre-1680 versus the post-1680 period (of the same length) despite the fading-record problem. This suggests that, in fact, many small fires did occur during the earlier (pre-1680 period).

Also, as previously noted, the East Fork site contained ancient tree-ring specimens extending well back into the 1300s CE. Sample sizes here remained relatively robust from the 2000s back to the 1500s, and the same fire occurrence patterns were observed, i.e. there were frequent small fires and relatively few widespread fires pre-1680, with more widespread fires after 1680 CE and until the livestock grazing/fire suppression era. Hence, we are confident that this general pattern is genuine, reflecting a high abundance of small fires and reduced widespread fires during the high-to-medium Jemez population density periods (i.e. 1300–1680 CE) in this portion of the Jemez Mountains.

Moreover, SEA results support a general conclusion that human land uses (timber harvesting, fuel wood use, trails, agriculture and purposeful/accidental setting of fires) probably overrode climate–fire associations in the pre-1680 period. After 1680 CE, with very reduced human populations and lack of extensive land uses, climate–fire associations were more strongly expressed. The wet/dry pattern typical of many semi-arid pine-dominant sites across western North America emerges as the common pattern in the Jemez Mountains only after 1680 CE. The specific role of human interference with climate–fire patterns within the Jemez Mountains is further indicated by SEA results using the much broader-scale records over the western North American network during similar time periods. This analysis shows no major change in interannual climate–fire associations when pre- and post-1680 periods are tested (results not shown).

Although the SEA results support an interpretation of human interference with interannual climate–fire associations before 1680 CE, it is possible that longer-term climate patterns could have played some role in changing fire patterns. Decades of the early 1600s, for example are commonly referred to as the early portion of the ‘Little Ice Age’. Estimated trends of biomass burning in western North America from sedimentary charcoal show decreased fire activity during the early 1600s and increased burning trends during the 1700s and 1800s [88]. Temperature-specific palaeoclimate reconstructions relevant to the south-west [89] do show the early decades of the 1600s had relatively cool summers during this period of infrequent widespread fires in the Jemez Mountains. However, decade to multi-decade temperature and fire trends in our study region were not consistent in sign over the whole period of analyses. For example, temperatures were generally near average or warm during the 1500s but few fires were widespread, and early decades of the 1800s were relatively cool again, but many widespread fires occurred.

The overall western North American network undoubtedly includes many forest stands, landscapes and fire regimes that were likewise strongly influenced by human land uses. The very broad (coarse-scale) compilation of hundreds of fire chronologies over this subcontinental area, however, shows that the classic interannual climate–fire association was the clearest general pattern prior to *ca* 1900. In particular, the wet/dry pattern was typical of the most extensive fire years (figures 2 and 3*a,b*). This result does not imply that humans were unimportant in altering fire regimes over western North America prior to the major Euro-American settlement era (i.e. 19th and early 20th centuries), but rather that the most pronounced effects were likely to be expressed at finer spatial scales and times. That is, human impacts on fire history are highly place- and time-specific. The most profound effects of human land use on fire regimes in western North America at the broadest scale are evident in the near complete cessation of widespread surface fires *ca* 1860–1900 within semi-arid ponderosa pine-dominant forests. This change coincided with, and was most likely associated with, the population declines and near complete removal of most Native Americans from ancestral lands, and their relocation onto reservations generally located in non-forested lowlands, arrival of railroads, the rise of intensive livestock grazing and fire suppression.

Given our broad- and fine-scale observations, we conclude that a generalized chronology of climate–human–fire interactions in relatively distinct time periods can be described for the south-west Jemez Mountains (electronic supplementary material, table S1). In summary, we label these periods and interpret their narratives as follows: (i) *Pre-colonial period* (pre-1590), characterized by relatively high human population densities in the uplands, intensive land uses (e.g. fuelwood and timber harvesting, agriculture, and trails) resulting in reduced fuel connectivity; essentially, no spreading fires near villages, but many small fires and very few widespread fires in more distant, seasonal agricultural areas or more distant areas; weak association of fire events with interannual climate variations; (ii) *Congregación* (1590–1680), characterized by depopulation of large village sites in the uplands; recovery of forests on village sites; increased mass and connectivity of fuels overall and a rise in number of widespread fires in some areas; weak association of fire events with interannual climate variations; (iii) *Free-range fire period* (1680s–1860s), characterized by open ponderosa pine-dominant forests with free-ranging, widespread surface fires (i.e. wildfires were not generally impeded by human actions) at decadal or subdecadal intervals; strong interannual climate–fire associations, especially with wet/dry oscillations related to large fire years; (iv) *Livestock grazing and fire suppression period* (1860s–present), characterized by intensive livestock grazing; leading to greatly reduced fine-fuel mass and continuity; subsequently, direct suppression of fires by government agents, leading to near elimination of widespread surface fires; timber harvesting, road building and lack of widespread fires leads to multiple cohorts of trees establishing, especially during wet periods; many homes and other structures built within forested areas (especially after World War II); fuel accumulations of live and dead trees increase and thickets of small-diameter, stunted pines become common; very large, high-severity wildfires occur during extreme drought years, especially after 1980 as temperatures rise.

These narratives emphasize the particulars of time and place in the Jemez Mountains, but we note several general patterns that relate to broader concepts relevant to fire regimes and humans. The high degree of sensitivity of fire regimes in the Jemez to fuel connectivity is illustrative of a potentially global characteristic of landscapes where fire occurrence and spread tends to be fuel-limited (i.e. semi-arid forests and woodlands). Archibald *et al*. [14] show similar effects of high human densities in southern Africa, where a combination of fuelwood use, livestock grazing and increased number of fire ignitions by people results in high frequencies of small fires of very limited extent, individually or cumulatively. This pattern contrasts sharply with an adjacent managed landscape (Kruger National Park), where human population densities are much lower, fires are allowed to spread only intermittently, and when they occur they are very widespread, burning large total areas. Archibald *et al*. [14] also show in model simulations that the effect of fuel connectivity in reducing (or enhancing) fire spread is nonlinear, with a threshold near 60%. That is, when fuel connectivity drops below this threshold (defined as continuous fuels between adjacent modelled cells of relatively fine spatial resolution) then adding many more ignitions or increasing the flammability of fuels (e.g. moisture content) have little effect on increasing the total area burned. The implications of these empirical observations and simulations then is that fire regimes can shift rather abruptly from landscapes with sufficient fuel connectivity that will sustain widespread fires, to landscapes with insufficient fuel connectivity to support spreading fires, regardless of how many additional local fires are started by people or how dry it gets [6,83]. The key controlling variable affected by people is fuel connectivity.

Overall, these results emphasize the importance of historical contingencies and geographical particularities on fire regime characteristics, hinging to a considerable degree on human population densities and land uses, and the timing of these human variables. Climate controls over fire activity are pervasive but can be swamped out by high-intensity land uses (and see an interesting and different example described in Australia, involving purposeful burning for hunting by the Martu people [90]). Furthermore, our results demonstrate that the commonly assumed effects of Native Americans on past fire regimes—namely, increased fire frequency—is an overly simplistic construct. The most significant impact of humans on fire activity in south-western US forests during the pre-modern era (before 1900 CE) and during the late 19th and 20th centuries was the reduction of widespread fires through the effects of land uses on fuels connectivity.

Debates over the appropriateness of forest management involving restoration of lower forest densities and the re-introduction of surface fires to mitigate wildfire problems would benefit from the broader perspectives of history, including long-time human land uses. The Jemez landscape is not unique in the south-west, or in other parts of North America, in having sustained significant human populations for multiple centuries within fire-prone forests prior to Euro-American settlement [15,91,92]. Our evidence indicates that intensive wood utilization and other practices resulted in a landscape with more heterogeneous (and lower-connectivity) fuels distribution than today. Intensive human land use in the past in these landscapes, with no evidence to date of occupied villages having burned over in past wildfires, suggests that judicious human management of forests today could be based at least in part on more detailed understanding of the importance of how climate and people interact to affect fire frequency and fuels.

## Supplementary Material

Supplementary material

## Data accessibility

Palaeoclimatic data used in this paper are accessible at the U.S. National Oceanic and Atmospheric Administration's Paleoclimatology database at http://www.ncdc.noaa.gov/paleo/pdsi.html.

Most of the tree-ring-based fire histories used included in this paper are deposited in the US National Oceanic and Atmospheric Administration's Paleoclimatology data base at https://www.ncdc.noaa.gov/data-access/paleoclimatology-data/datasets/fire-history.

Further information on these datasets are at www.firescience.gov/projects/09-2-01-10/project/09-2-01-10_final_report.pdf.
