# Supplementary material for: Multiscale perspectives of fire, climate and humans in western North America and the Jemez Mountains, USA
Source: Philos Trans R Soc Lond B Biol Sci. 2016 Jun 5;371(1696):20150168. doi: 10.1098/rstb.2015.0168 (PMC4874406; doi:10.1098/rstb.2015.0168)
Supplement: Supplementary material [file rstb20150168supp1.pdf]

Multi-Scale Perspectives of Fire, Climate and Humans in Western North America and the Jemez Mountains, U.S.A.

Thomas W. Swetnam, Joshua Farella, Christopher I. Roos, Matthew J. Liebmann, Donald A. Falk & Craig D. Allen

Supplementary Material

Table S1 describes generalized descriptions of interpreted human land uses, forest structures and fire regimes in the Jemez Mountains during different time periods and areas that sustained relatively high, medium and low human population densities. The exact dates of the time periods selected are somewhat arbitrary in that the timing of human population changes as well as impacts on forests and fire regimes varied substantially from place to place. The Pre-Colonial Period, for example, is listed as before 1590, whereas the first major colonization of New Mexico was in 1598 by settlers led by Juan de Oñate, and earliest Jemez missions were also established near that time [1,2]. However, earlier Spanish explorers had entered New Mexico in previous decades. It is possible that European diseases preceded Onate's settlers in New Mexico, but there is a lack of any certain evidence for effects on Jemez populations until the early 1600s. In any case, we chose 1590 as a convenient break point and an earliest time when populations might have been affected by effects of colonialism. Also, human population declines and de-population of large village sites in the Jemez mostly occurred from circa 1620s to 1650s [2].

Likewise, the 1860 date for the advent of livestock grazing is somewhat earlier than the timing of large herds arriving in most of the interior portions of the Jemez Mountains. Kit Carson and the U.S. Army subdued the Navajos in 1863, but they remained a threat to safety in the Jemez Mountains for some decades after that [3], and so livestock grazing in most areas within the Mountains was very limited until the 1870s or later. However, the Jemez fire scar chronology network spans the greater Jemez Mountains, including forests and woodlands on the far northern and eastern flanks of the Mountain (in the Chama River and Rio Grande Watersheds, respectively), and in these areas early livestock grazing and impacts on fire extent likely occurred by the mid-1800s and 1860s [4]. The composite Jemez Mountains fire chronology (Figure 4A-B) reflects the early 1860s disruption of widespread fires, while the finer scale stand examples from the interior of the Mountains at Monument Canyon and East Fork sites shows later widespread fire disruptions in the 1870s to 1900 (Figures 4B-D, S4, S5). Hence, the break points (dates) used in the periods in Table S1 reflect a trade-off in specificity for generality, as well as for the purpose of using sufficiently long and similar length periods for our comparisons of fire frequency changes (e.g., 1500-1680, and 1681-1860, Table 1).

Table S1. Generalized human, forest and fire chronology narratives for the Southern Jemez Plateau, New Mexico, circa 1300 CE to Present. Land uses are first described for each time period, and then forest and fire regime characteristics are described for each intensity level of land use: high, near villages; moderate, agricultural and field house areas; low, distant from villages and agricultural areas.

**Generalized Period Name:**	**Pre-Colonial Before 1590CE**	**Congregación 1590-1680 CE**	**Free Range Fire 1680s-1860s CE**	**Livestock Grazing & Fire Suppression 1860s-Present**
**Human Land Uses:** **General Patterns on southern Jemez Plateau**	Extensive fuel wood gathering, timber harvesting, trails and agricultural fields; human settlements primarily in uplands on mesas; some fire ignitions added by people purposefully (e.g., for agriculture, hunting, pilgrimage, or other uses) or accidentally.	Reduced fuel wood gathering in upland areas; decreased human populations; decreased agriculture & travel in upland areas; increased fuel wood gathering in lowland areas near pueblos & missions; increased farming & irrigation in lowland areas; some fire ignitions added by people purposefully (e.g., for agriculture, hunting, pilgrimage, or other uses) or accidentally.	Near complete de-population of uplands (except during Revolt/Re-Conquest period, 1680-1696); some seasonal and transient use continues; some traditional trails still used, but mainly seasonally for hunting and ceremonial purposes; relatively few fire ignitions by people compared to early periods.	Intensive livestock grazing begins in most areas; peak grazing 1880s-1910s; US Forest Service begins fire suppression efforts, especially after 1910, and combined with livestock grazing the effect is near complete elimination of widespread surface fires; extensive road building and timber harvesting begins in 1920s and continues through 1970s; many Hispanic and Anglo-American structures are built within forest areas, especially post WWII; many fire ignitions by people.
**Forest Structure (Fuels)/Fire Regimes:** **Near Villages:**	Sparse ground vegetation present on upland mesas, widely scattered trees in very open forest stands; fuel connectivity disrupted by trails and fuel wood/timber harvesting; no surface fuels or canopy fuel continuity within and near villages, and so no spreading fires; occasional small fires in cliff escarpments near villages, but fire events uncorrelated with inter-annual climate variability (Farella 2015).	Most upland villages are de-populated and vegetation recovery begins in these areas; some fuels continuity re-establishes with grass and forb cover and with some tree canopy coverage and needle cast accumulating; fires begin to occur on and near village sites within 10 to 30 years of de-population; fire occurrence generally uncorrelated to inter-annual climate variations until after 1680 CE.	Forest/vegetation recovered on most depopulated villages and fields by ca. 1700 CE; open, park-like stands develop and are maintained by frequent (<20 yr interval) surface fires; most trails also grown over, and covered in grasses or pine needles; continuous surface fuels present wherever soils and inter-annual/decadal climate conditions allow, and where fires have not burned for 1 to 5 years; fire occurrence strongly correlated with inter-annual climate variability, especially wet/dry oscillations.	Livestock grazing, drive-ways and trails result in extensive dis-continuity of surface fuels; logging and road building lead to removal of old growth forest structures, and subsequent extensive regeneration of trees during wet periods, especially in the absence of spreading surface fires; by mid-20th century there are extensive live and dead fuel accumulations, and thickets of stunted conifers are common, creating closed/continuous forest canopies; large, high severity wildfires occur with increasing frequency after circa 1980 during extreme drought years.

**Forest Structure (Fuels)/Fire Regimes:** **Seasonal Agricultural/ Fieldhouse Areas**	Forests are generally open and park-like, but less dense than during the subsequent periods due to long-term fuel wood use and timber harvesting; agricultural fields and plots in large canopy openings and trails disrupt fuel continuity; surface fuels are occasionally continuous enough to allow spreading fires; small fires (non-synchronous among trees and sites) are fairly common; fire events are generally uncorrelated with inter-annual climate variability.	Surface fuels become more continuous as villages are depopulated and trails and fields fall out of use; some fields in the uplands are still in use 1640-1690s CE, and short term occupation/re-occupation of village sites during years/periods of fighting and unrest during Revolt and Reconquista Periods (1680s-1690s resulting in short term fuel dis-continuities; a few more widespread fires occur than before 1640 CE; fire occurrence generally uncorrelated to inter-annual climate variations until after 1680 CE.	Open forest canopies with grass and pine needle cover become most typical of upland landscapes (below about 9,000 ft); and fire occurrence similar to Near Villages sites, i.e., frequent surface fires; fire occurrence strongly correlated with inter-annual climate variability, especially wet/dry oscillations.	See above; similar to Near Villages in most described aspects.
**Forest Structure (Fuels)/Fire Regimes:** **Distant from Villages & Agricultural Areas**	See above, similar to field house and agricultural areas, but less pressure from fuel wood or timber harvesting, and trails, so the surface fuels are relatively more continuous than in agricultural areas; many small fires (non-synchronous between trees) occur, due to ignitions by both people and lightning; few widespread fires because of heterogeneous fuels related to very frequent small fires; fire events are generally uncorrelated with inter-annual climate variability.	See above; similar to Near Villages and to Near/Within Fieldhouse & Agricultural Areas, with mostly small fires and occasional or rare widespread fires; fire occurrence generally uncorrelated to inter-annual climate variations until after 1680 CE.	See above; similar to Near Villages and to Near/Within Field House & Agricultural Areas in most described aspects.	See above; similar to Near Villages and to Near/Within Field House & Agricultural Areas in most described aspects.

Figure S1. Fire-scarred trees record the history of forest fires within their tree rings. At left, a low severity surface fire re-burns within a fire scar cavity created by previous fires. Pitch and resin in the wound and exposed wood readily re-ignite from even low intensity surface fires. At right is a typical cross section of a fire scarred ponderosa pine tree from the Jemez Mountains, New Mexico. Commonly, fire scarred pines from this mountain range have 10 to 20 scars over a period of 300 or more years. The last widespread fire recorded by most trees was in the late 1800s when intensive livestock grazing began.

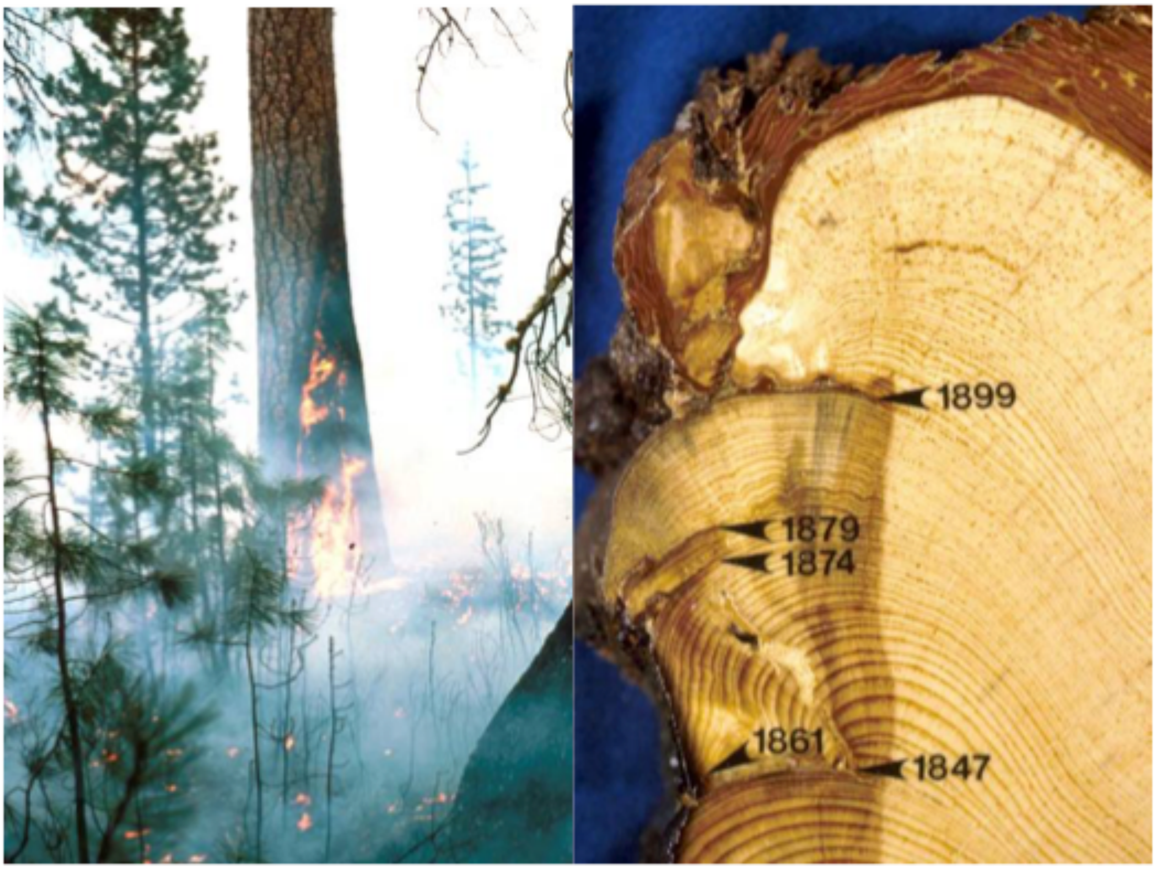

Figure S2. The Southern Jemez Plateau in northern New Mexico sustained 5,000 to 8,000 people living within a 180,000 ha forested area from circa 1400 to 1620 CE. At least 10 villages had 500 to 1,500 rooms each, and 3 to 4 story stone masonry buildings. The surrounding landscape today is forested with ponderosa pine, pinyon pine and juniper trees. Aerial photography (lower left) and LiDAR surveys (lower right) reveal the outlines of room blocks of the large village ruins within pine forests. We sampled living trees growing within and near the ruins in four village sites to refine dates of likely depopulation, and to learn the history of forest regeneration and fires burning in the vicinity (see Figure S3, [2,5])

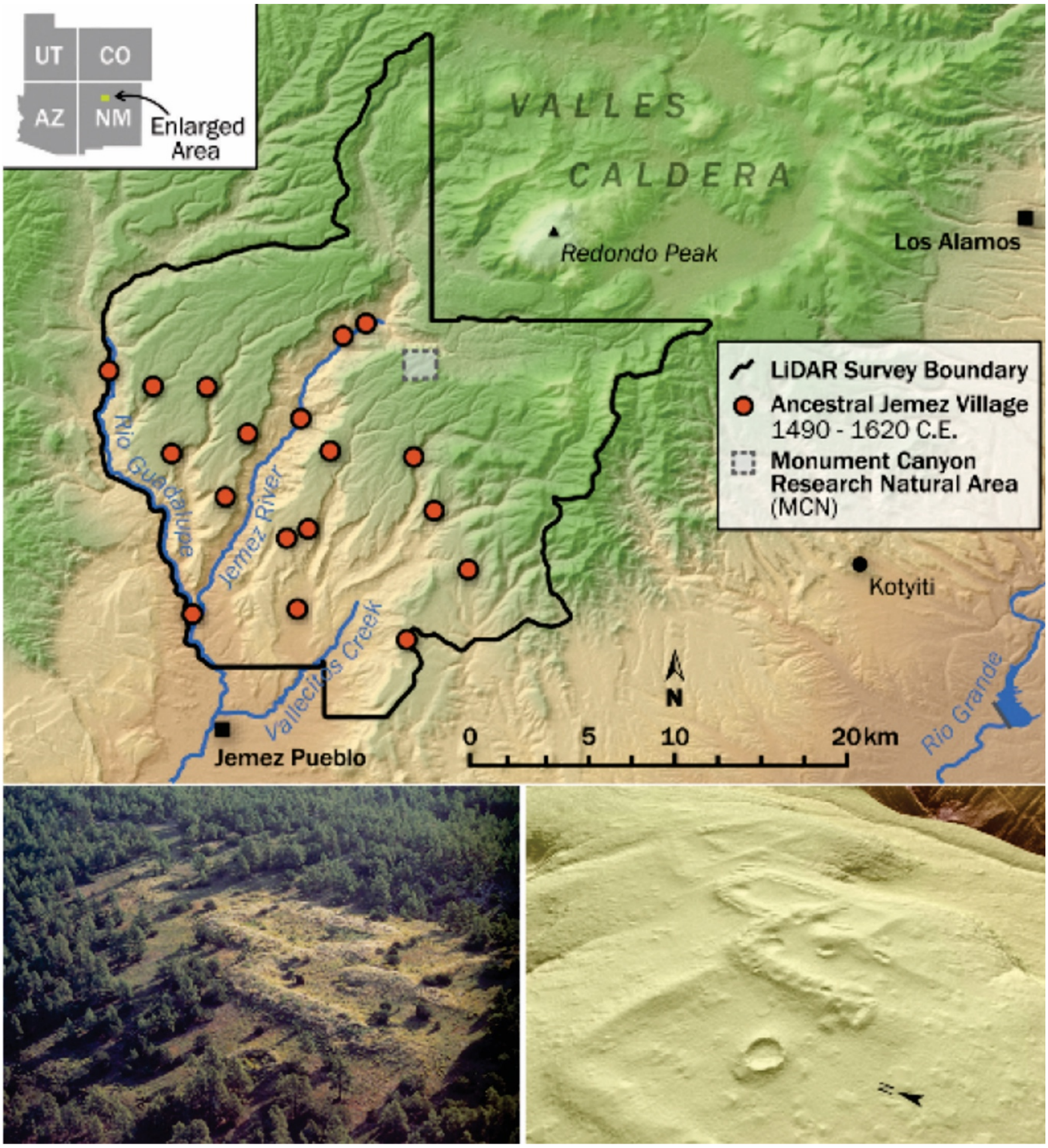

Figure S3. By sampling and tree-ring dating trees that established on village ruins following depopulation we estimated the timing of the last major use of the village sites, i.e., via the *terminus ante quem* dating method [5]. The village of Tovakwa is shown (upper left) with symbols indicating the earliest dates of tree establishment. All trees near and within the village established after 1650 CE, as shown by the chart at the bottom. This indicates likely departure of most or all people 5 to 20 years earlier, as it would take some time for soils to become loosened and tree seedlings to become established. Scattered, ancient tree remnants with a few fire scars were also found at this site in the rocky cliffs to the southeast of the village (see the map in upper left, and tree-ring dates labeled on the chart at bottom). Small (single tree) fire events (labeled on the Lidar image) in some of these trees suggests that there was occasional burning near the village during occupation. Fires were unlikely to ignite or spread very near or within the village because of lack of trees and fuels. After depopulation circa the 1640s CE fires began to be recorded on the trees that established there (at bottom).

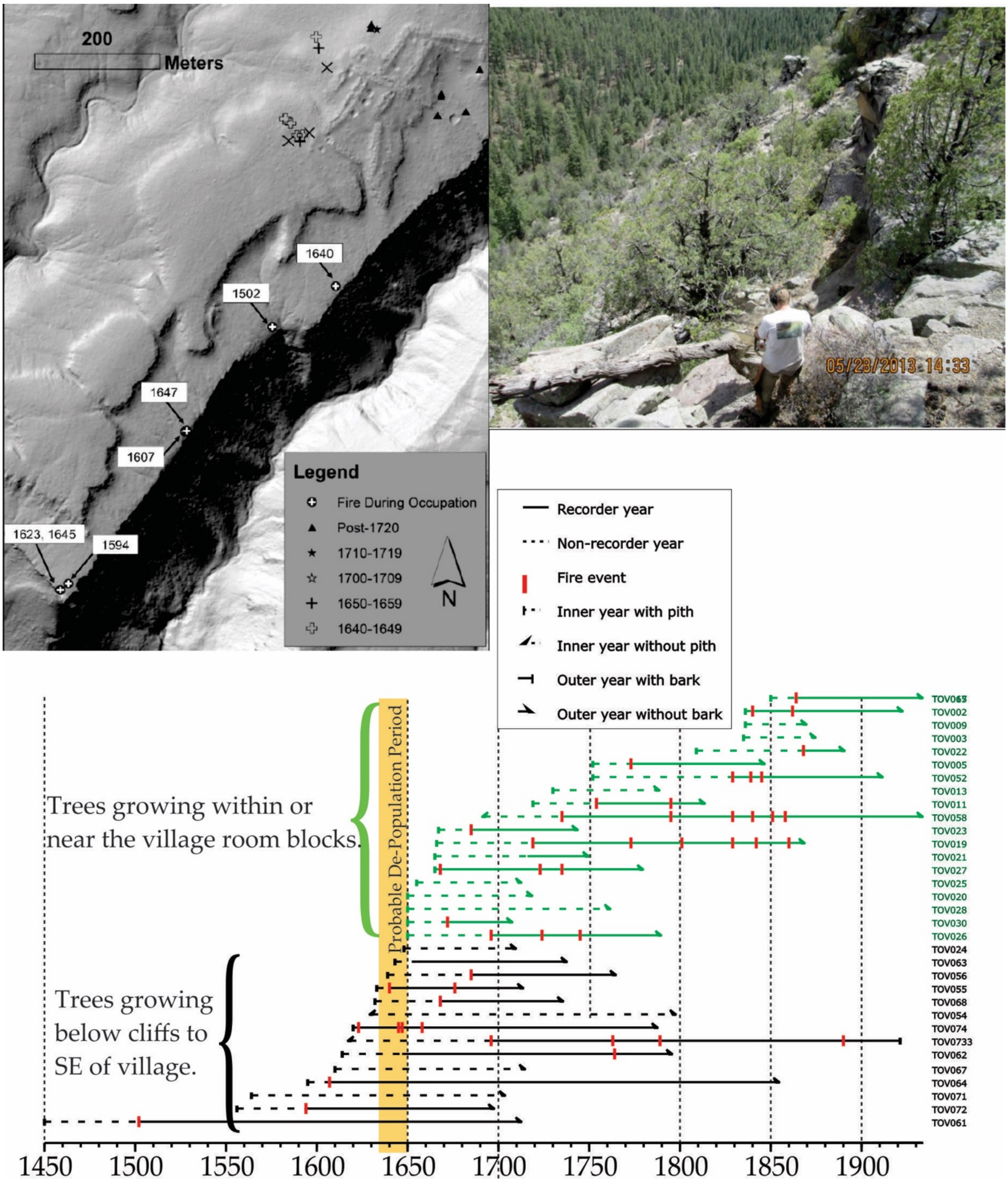

Figure S4. The fire chronology from 192 trees at the 260 ha Monument Canyon Research Natural Area illustrates the temporal changes in fire regime in an area associated with seasonal "field house" occupation and agricultural use. Individual lines are composites from 2 to 10 sampled fire scarred trees from grid points systematically and randomly spaced across this study area [6]. The vertical lines at the bottom show the occirrence of a fire scarring any tree (All Fires), and widespread fires recorded by 25% or more of the grid points.

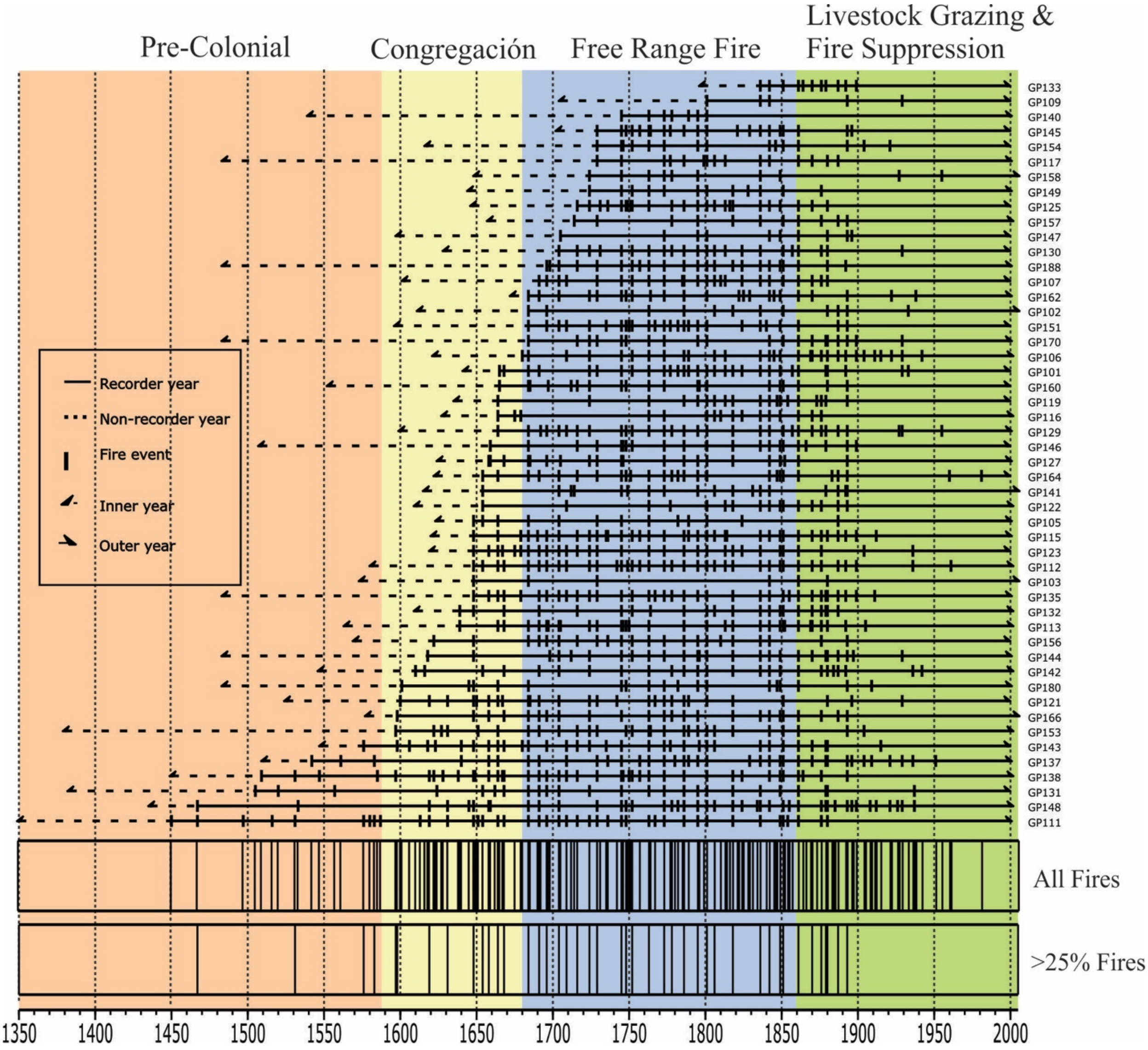

Figure S5. The East Fork fire scar chronology, comprised of 26 trees collected in about a 5 ha area that is distant from villages and agricultural sites. This chronology illustrates the same pattern of frequent, small fires prior to the 1680 Pueblo Revolt, but relatively few widespread fires in comparison to the post-1680 period.

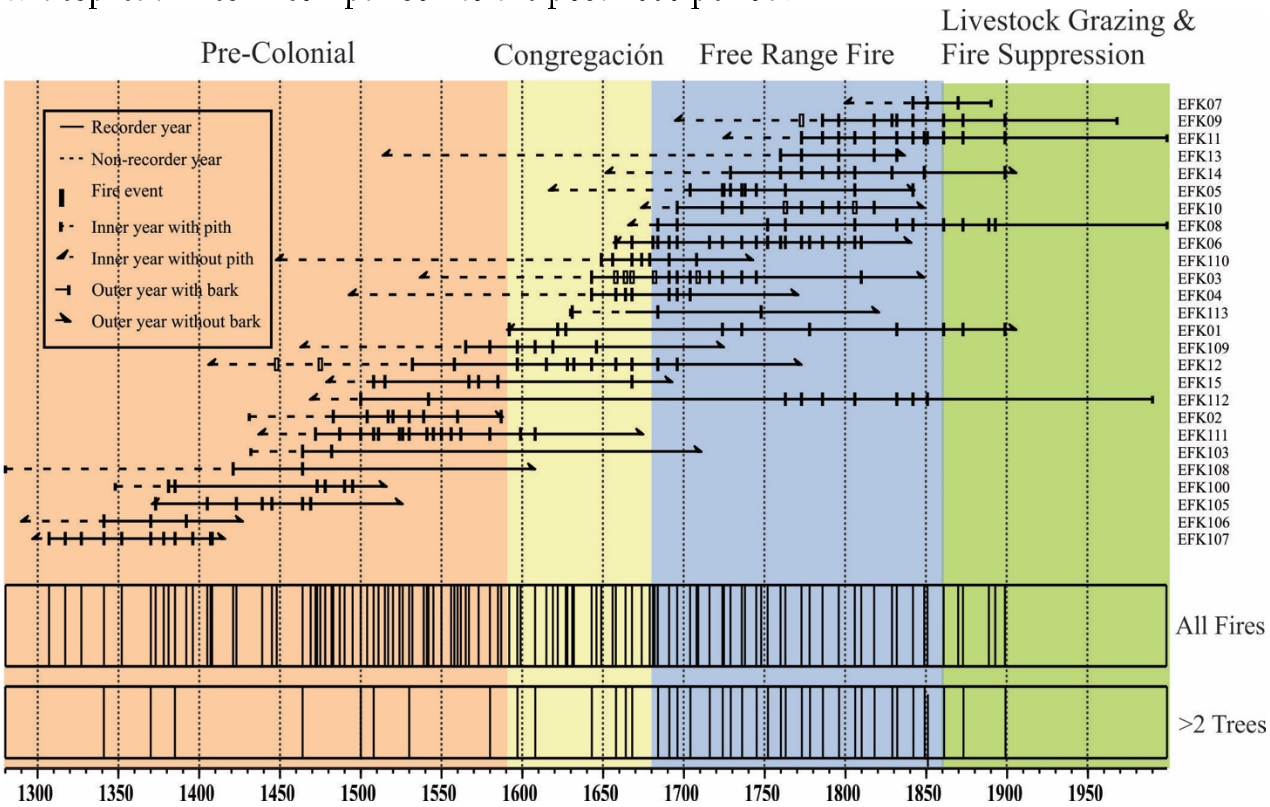

Figure S6. Sampling curves showing mean fire frequency as a function of simulated increasing sample sizes in the Jemez Mountains (n = 1,377 trees), Monument Canyon (n = 198 trees) and East Fork (n = 26 trees) sites, as determined using the FHAES program [7,8]. The sample size analysis algorithm uses a bootstrap resampling without replacement of specimens within a dataset to estimate mean fire intervals (frequency, fires/century) at each potential sample size up to the full size of the data set (i.e., a sampling curve). The curves shown are for the later, 1681-1860 period. Filled circles show fire frequency in the earlier time period (1500-1680) for comparison, with frequencies (the circles) plotted on the median sample size during the 1500-1680. The horizontal lines plotted with the filled circles show the range of sample sizes during the earlier period.

The East Fork shows the strongest pattern, with fewer widespread fires (fires recorded on 2 or more trees) during the pre-1680 period than the later period, even when accounting for sample size effects. In contrast, there were more fires detected overall (any fire) in the pre-1680 period than later. MCN shows a similar pattern, but with a less change in fire frequency of the "all fires" case (depending on sample size, which changed a great deal from 1500 to 1680, i.e., from 8 to 130 specimens).

All scales of analyses show that there were more single tree fire events recorded in the earlier than in the later period.

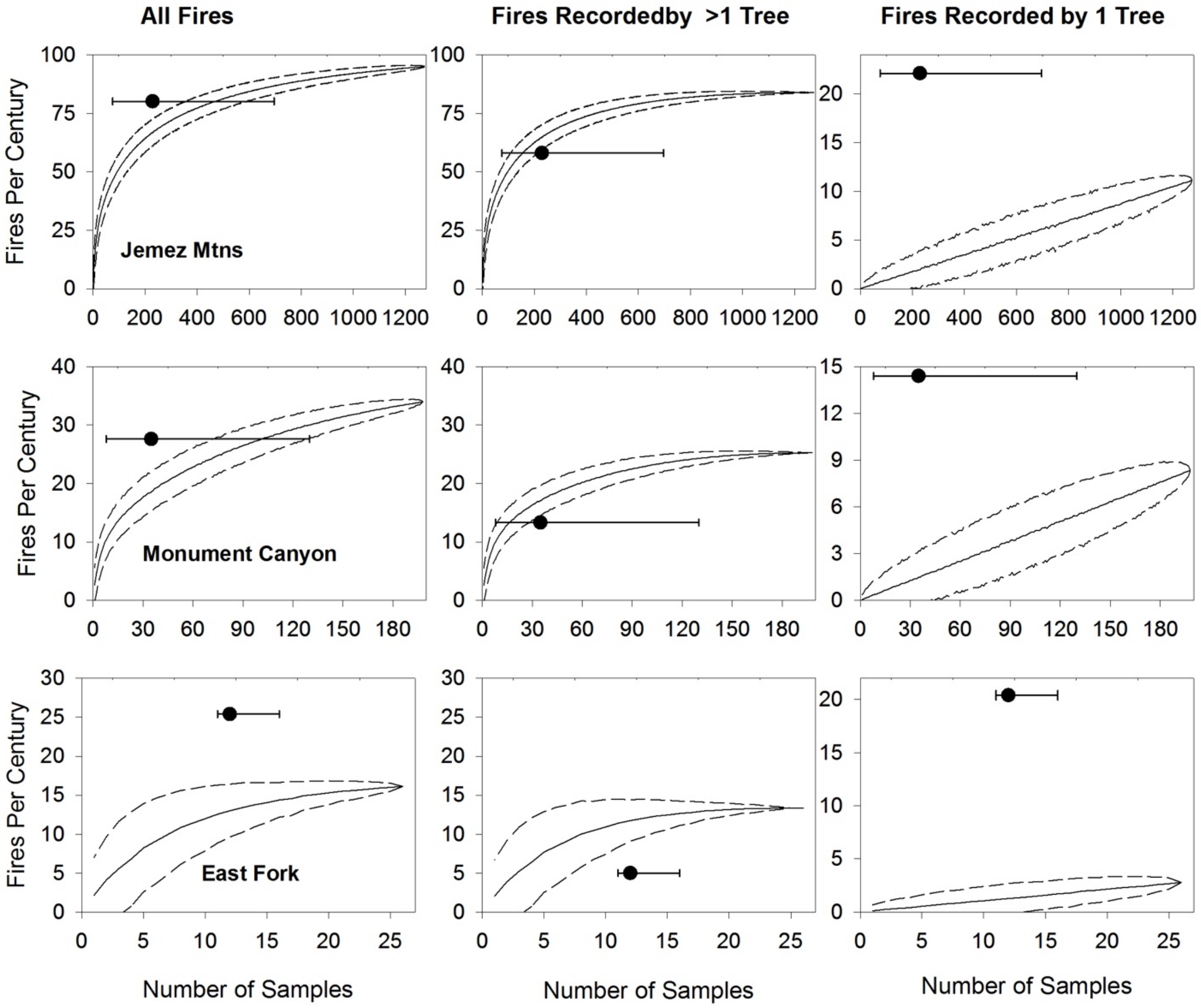

References Cited

1. Liebmann M. 2012 Revolt: *An archaeological history of Pueblo resistance and revitalization in 17th century New Mexico*. Tucson, Arizona, U.S.A.: University of Arizona Press.

2. Liebmann MJ, Farella J, Roos CI, Stack A, Martini S, Swetnam TW. 2016 Native American Depopulation, Reforestation, and Fire Regimes in the Southwest U.S., 1492-1900 C.E. *Proc. Natl. Acad. Sci. U.S.A.* In Press

3. Anschuetz KF, Merlan T. 2007 More than a scenic mountain landscape: Valles Caldera National Preserve land use history. *U.S. Forest Service General Technical Report RMRS-GTR-196*. Fort Collins, Colorado, U.S.A.: U.S. Department of Agriculture, Forest Service, Rocky Mountain Research Station.

4. Touchan R, Swetnam TW, Grissino-Mayer HD. 1995 Effects of Livestock Grazing on Pre-Settlement Fire Regimes in New Mexico. pp 269-272 In *Proc. Symposium on fire in wildreness and park management, 1993 March 30-April 1, Missoula, Montana, U.S. Forest Service General Technical Report INT-GTR-320* (eds JK Brown, RW Mutch, CW Spoon, RH Wakimoto). Ogden, Utah, U.S.A.: U.S. Department of Agriculture, Forest Service, Intermountain Mountain Research Station.

5. Farella J. 2015 Terminus ante quem constraint of pueblo occupation periods. Master of Arts Thesis, Department of Geography and Regional Development, University of Arizona.

6. Falk DA, Swetnam TW. 2003 Scaling rules and probability models for surface fire regimes in ponderosa pine forests. pp 301-318 In *Fire, Fuel Treatments, and Ecological Restoration, U.S Forest Service Proceedings RMRS-P-29* (eds PN Omi, LA Joyce). Fort Collins, Colorado U.S.A.: U.S. Deapartment of Agriculture Forest Service Rocky Mountain Research Station.

7. Brewer PW, Velásquez ME, Sutherland EK, Falk DA. 2015 Fire History Analysis and Exploration System (FHAES) version 2.0.0, [computer software], http://www.fhaes.org. (doi:10.5281/zenodo.34142)

8. Sutherland EK, Brewer PW, Falk DA, Velásquez ME. 2016 Fire History Analysis and Exploration System (FHAES) user manual [compiled 02/11/2015] http://www.fhaes.org.
